# Supplementary material for: Integrated Analyses of Single-Cell Transcriptome and Mendelian Randomization Reveal the Protective Role of Resistin in Sepsis Survival in Intensive Care Unit
Source: Int J Mol Sci. 2023 Oct 7;24(19):14982. doi: 10.3390/ijms241914982 (PMC10573869; doi:10.3390/ijms241914982)
Supplement: Supplementary file 1 [file ijms-24-14982-s001.zip › Supplementary Figures S1 and S2.pdf]

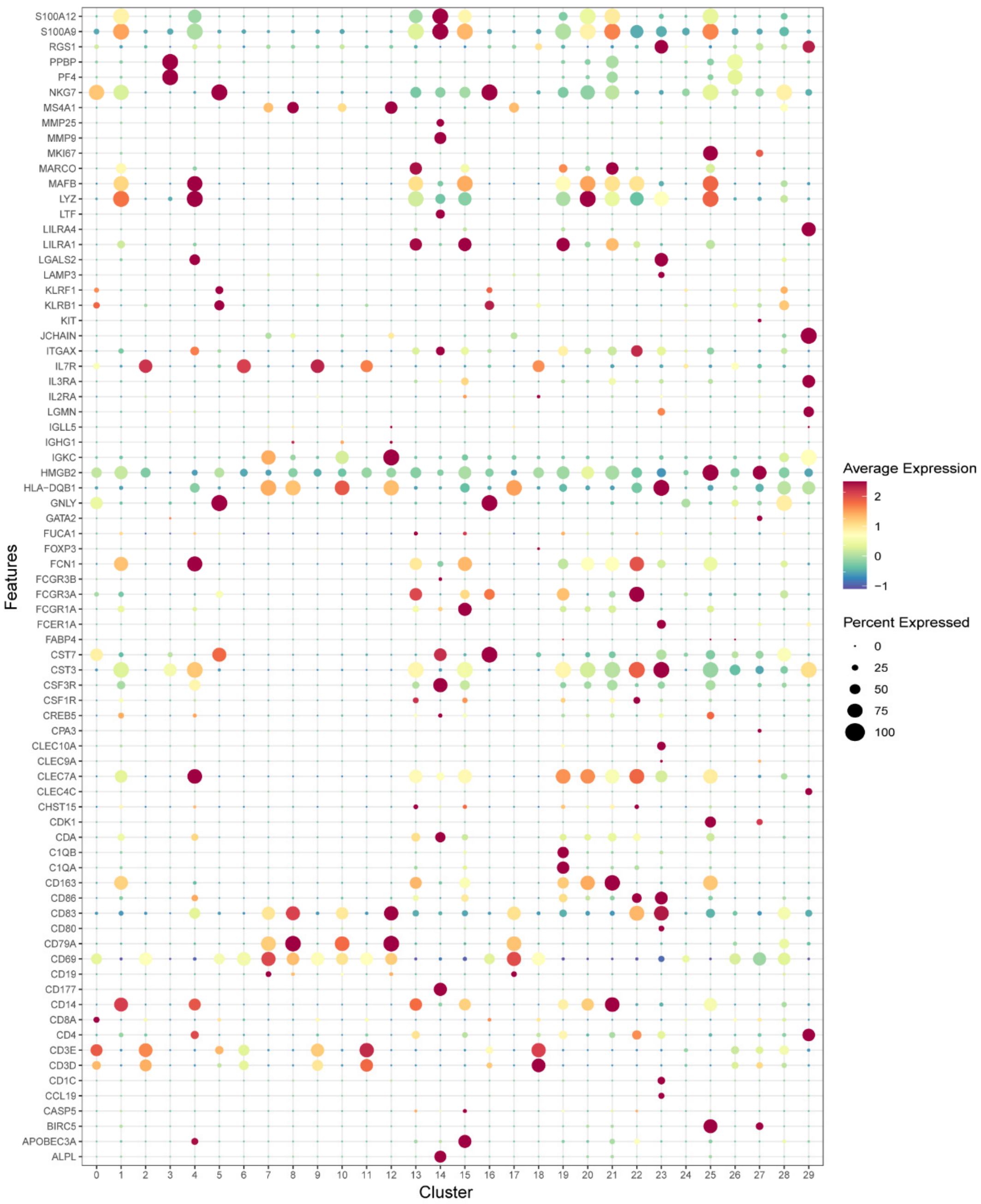

Figure S1. The dotplot shows the average expression levels and ratios of all cell markers in 30 clusters.

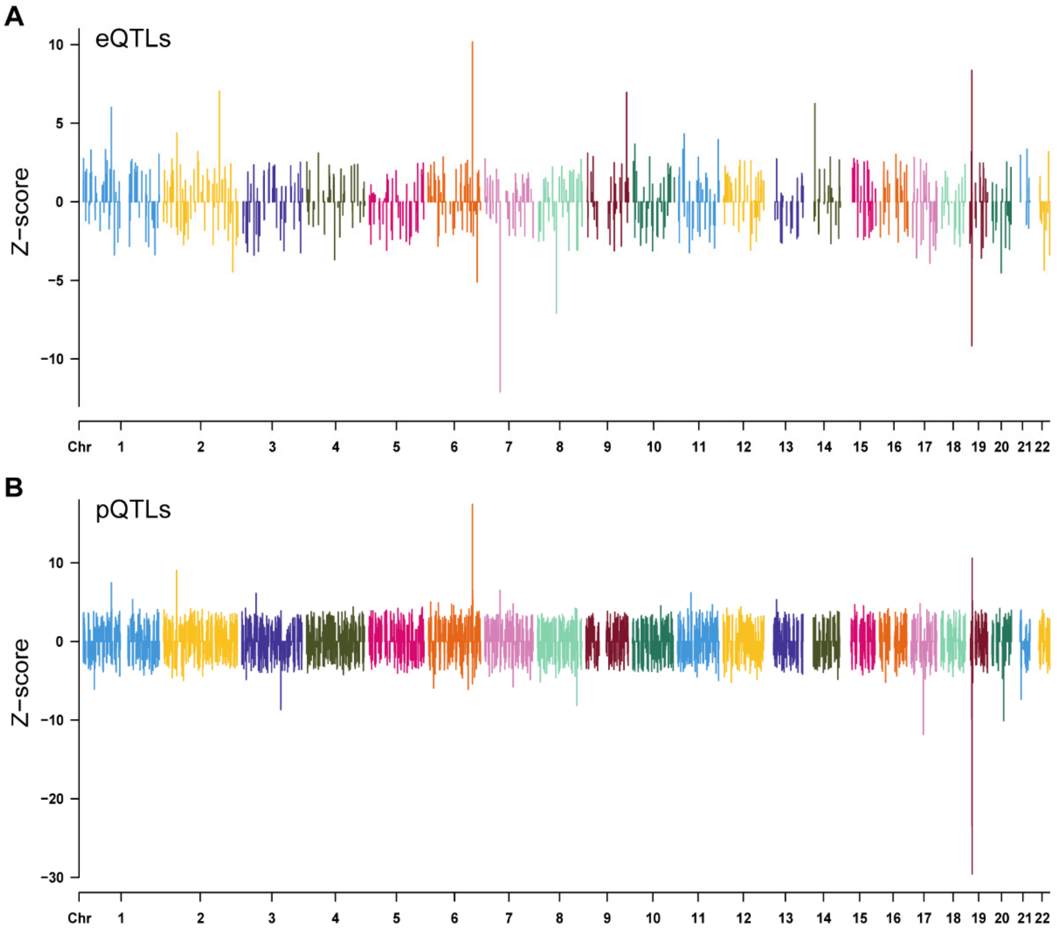

Figure S2. The Z scores of IVs. **A-B** The Z scores of eQTLs (**A**) and pQTLs (**B**). Z score represents the scaled direction and efficiency of association between SNP and RETN.
