# Supplementary material for: Integrated Analyses of Single-Cell Transcriptome and Mendelian Randomization Reveal the Protective Role of Resistin in Sepsis Survival in Intensive Care Unit
Source: Int J Mol Sci. 2023 Oct 7;24(19):14982. doi: 10.3390/ijms241914982 (PMC10573869; doi:10.3390/ijms241914982)
Supplement: Supplementary file 1 [file ijms-24-14982-s001.zip › Table S1. All statistical values about QTLs of RETN.pdf]

Table S1 All information about the proxy SNPs of resistin (eQTLs, pQTLs).

QTL type	SNP	Effect allele	Other allele	EAF	*p*	Beta	SE	F
eQTLs	rs34856868	A	G	0.025	2E-09	0.231	0.038	36.076
	rs1375493	A	G	0.565	2E-12	0.084	0.012	49.445
	rs149110519	T	C	0.053	3E-24	0.270	0.027	103.435
	rs149007767	T	C	0.150	1E-33	-0.201	0.017	146.071
	rs7846314	T	A	0.178	1E-12	-0.110	0.016	50.138
	rs13289095	T	G	0.120	3E-12	0.128	0.018	48.497
	rs2239630	G	A	0.519	4E-10	0.074	0.012	38.921
	rs1423096	C	T	0.910	5E-20	-0.190	0.021	83.893
	rs34124816	C	A	0.027	1E-18	-0.321	0.036	77.752
	rs35547567	T	C	0.012	6E-17	0.453	0.054	69.981
	rs4804793	A	G	0.969	1E-16	-0.285	0.034	68.550
pQTLs	rs3917932	G	C	0.431	1E-09	-0.0531	0.009	37.022
	rs141094656	C	T	0.028	9E-14	0.1954	0.026	55.686
	rs2074038	T	G	0.092	6E-10	0.0919	0.015	38.386
	rs11078930	T	C	0.375	4E-32	-0.105	0.009	139.343
	rs117916280	A	T	0.035	6E-23	-0.2357	0.024	97.412
	rs137964046	A	G	0.014	7E-44	-0.5023	0.036	192.972
	rs117029024	A	G	0.033	1E-122	-0.5646	0.024	554.673
	rs35547567	T	C	0.008	4E-26	0.5362	0.051	111.992
	rs3745368	A	G	0.039	7E-192	-0.6658	0.023	873.148
	rs183703689	A	G	0.006	2E-22	-0.5277	0.054	94.542
	rs13390874	T	C	0.246	1E-19	0.0907	0.010	81.888
	rs112694524	A	G	0.050	5E-11	0.1306	0.020	43.079
	rs4811689	A	G	0.458	7E-24	-0.0872	0.009	101.531
	rs243596	A	G	0.487	2E-13	-0.0636	0.009	54.286
	rs56185965	A	T	0.159	1E-09	0.0712	0.012	37.390
	rs150918492	A	G	0.292	5E-18	-0.0821	0.009	75.065
	rs1144700	T	C	0.180	4E-09	-0.0668	0.011	34.786
	rs547211157	C	G	0.020	1E-09	-0.192	0.032	37.032
	rs149110519	T	C	0.048	4E-68	0.3487	0.020	304.040
	rs138604055	A	C	0.016	6E-11	0.2196	0.034	42.665
	rs2168589	A	T	0.319	8E-11	0.0599	0.009	42.318
	rs445	T	C	0.090	8E-09	-0.0885	0.015	33.354
	rs10103048	C	A	0.390	3E-16	-0.072	0.009	66.654
