# Supplementary material for: Integrated Analyses of Single-Cell Transcriptome and Mendelian Randomization Reveal the Protective Role of Resistin in Sepsis Survival in Intensive Care Unit
Source: Int J Mol Sci. 2023 Oct 7;24(19):14982. doi: 10.3390/ijms241914982 (PMC10573869; doi:10.3390/ijms241914982)
Supplement: Supplementary file 1 [file ijms-24-14982-s001.zip › Table S2. Results of sensitivity analyses.pdf]

Table S2. Sensitivity analyses for main results of Mendelian randomization.

IEU ID (outcome)	Trait	Proxies of RETN	Heterogeneity test (Q)	Pleiotropy test (*p*)	Directionality test (*p*)
ieu-b-4981	Sepsis (28 day death in critical care)	eQTLs	0.782	0.240	4.65E-163
		cis-eQTLs	0.098	0.867	4.87E-84
		pQTLs	0.313	0.352	0
		cis-pQTLs	0.590	0.521	0
ieu-b-4982	Sepsis (critical care)	cis-eQTLs	0.400	0.917	6.52E-86
		pQTLs	0.569	0.941	0
		cis-pQTLs	0.402	0.151	0
